# Impact of growth factor content on proliferation of mesenchymal stromal cells derived from adipose tissue

**DOI:** 10.1371/journal.pone.0230265

**Published:** 2020-04-16

**Authors:** Katrin C. Franz, Christoph V. Suschek, Vera Grotheer, Mehmet Akbas, Norbert Pallua

**Affiliations:** 1 Department of Plastic and Reconstructive Surgery, Hand Surgery and Burn Centre, Medical Faculty, Pauwelsstrasse, Aachen, Germany; 2 Department for Orthopaedics and Trauma Surgery, Medical Faculty, Heinrich-Heine-University Düsseldorf, Moorenstrasse, Düsseldorf, Germany; 3 Arteo Clinic for Plastic and Aesthetic Surgery, Johannstrasse, Düsseldorf, Germany; Università degli Studi della Campania, ITALY

## Abstract

Autologous adipose tissue (AT) transfer has gained widespread acceptance and is used for a broad variety of regenerative clinical indications. It is assumed that the successful outcome of AT transfer essentially depends on the amount of autocrine-generated growth factors (GF). It is supposed that several GF enhance and improve the anatomic and functional integration of the transplanted AT grafts at the site of implantation. In the present study we have investigated for the first time the correlation between the concentration of GF of freshly isolated AT and the proliferation and migration capacity of mesenchymal stroma cells (MSCs) derived from the respective AT sample. We here show that the proliferation and migration capacity of MSCs strongly depends on the GF content of the AT the cells were isolated from but in an inversely proportional manner. The lower the GF content of an AT sample was, the higher was the proliferation and migration capacity of the respective MSC population contained in the AT and *vice versa*. Furthermore, we found that supplementation with recombinant GFs only in the case of AT samples with low but not with higher growth factor contents led to a significant enhancement of proliferation and migration of the AT-resident MSCs. As we further show, this inefficiency of GFs to enhance MSC proliferation and migration in AT samples with high GF contents indicates a GF-mediated negative feedback mechanism leading to an impaired GF signaling in MSC obtained from those AT samples. Our results might explain why the successful use of AT grafting is frequently limited by low and unpredictable survival rates, and we suggest to use the knowledge of GF content of harvested AT as a predictive clinical parameter for risk assessment of the therapeutic outcome of autologous AT transfer.

## Introduction

Mesenchymal stromal cells (MSCs) represent an effective reservoir for various growth factors and regenerative molecules. By their ability to migrate to the site of a tissue damage MSCs have an extraordinary therapeutic potential in regenerative medicine. Various therapeutic approaches are based on the systematic administration of previously isolated MSCs. The success of this application form of MSCs depends essentially on the fact that the greatest possible number of these cells migrate successfully into the desired tissue area after their administration. Unfortunately, only a small percentage of the cells reach the target tissue after systematic administration. Therefore, current efforts concerning the improvement of the therapeutic efficiency of MSC-based therapies, spatially address the molecular mechanisms underlying MSC homing, i.e. initial tethering of MSCs by selectins, activation by cytokines, arrest by integrins, diapedesis or transmigration, their extravascular migration toward chemokine gradients (for review see [1]).

In modern reconstructive surgery, attempts are also made to use the positive effects of MSCs, with isolated MSCs being used directly locally or even being transplanted into the lesion area in their natural tissue environment. The transplantation of adipose tissue has proven particularly useful here. Adipose tissue is an ideal material for use as a permanent soft tissue replacement [2–4]. It has found wide acceptance and is used for a variety of indications [5–8]. It is readily obtainable, easy to sculpture, and as it is autologous, the risk of antigenic and allergic reactions is minimized. Thus, autologous adipose tissue transfer is a reliable and safe method of soft-tissue augmentation. Nevertheless, the knowledge about graft behavior after transplantation remains inadequate. Especially the variation of long-term outcome and loss of transplanted volume represents a well-known disadvantageous phenomenon [9–12]. There are several theories trying to explain this volume loss [13, 14]. A plausible explanation for volume reduction is insufficient blood supply to the implanted adipose tissue at the site of transplantation. This might result in increased apoptosis and necrosis of the transplanted mature adipocytes due to their low tolerance to the potentially ischemic environment as well as slow vascularization rate [15–17]. It is generally accepted that successful angiogenesis and neovascularization are essential for adipocyte survival and achieving a stable therapeutic outcome [18]. It has been shown that under normoxic conditions adipocytes and adipose tissue-derived stroma cells (ASCs) can survive longer than a week. However, after grafting of adipose tissue, adipocytes viability dramatically decreases and survive only 12 hours, whereas ASCs can survive 72 hours and viability rapidly decrease thereafter [19, 20]. The plasmatic nutrient supply of adipocytes by diffusion takes place at a maximum tissue depth of 1.5 mm [21]. Under conditions of diffusion-dependent supply only 40% of adipose tissue survives at a depth of 1.5 mm [22].

Angiogenesis depends directly on the presence of suitable growth factors. Already after a short latency period, the germination vessels organize into a functioning capillary blood supply network that allows the survival of adipocytes [23]. Important trigger proteins of angiogenesis are the angiopoietins, which are secreted by adipose tissue depending on the status of differentiation and external stimuli. Growth factors also contribute to cell proliferation [24]. Thus, *basic fibroblast growth factor* (bFGF), *vascular endothelial growth factor* (VEGF), and *insulin-like growth factor* (IGF) synergistically improve the growth of adipose tissue [25]. Growth factors substantially affect the stromal vascular fraction in the surrounding tissue [26] by enhancing vascularization and replacing fibrotic tissue with more supple tissue. In addition, growth factors promote precursor cell differentiation and neoangiogenesis thus enabling better volumetric results. More importantly, bFGF, IGF, and VEGF are tyrosine kinase receptor–mediated growth factors that have been shown to improve transplantation results in animal studies. Thus, bFGF enhances the migration and proliferation of endothelial cells [27, 28] and thus represents a strong mitogenic factor for adipocytes [29]. IGF-1 significantly improves the results of autologous transfer of adipose tissue and survival of adipocytes [30]. Finally, VEGF promotes endothelial proliferation and migration, and substantially increases angiogenesis [31, 32]. Due to these findings, it had been assumed that preconditioning of adipose tissue transplant with growth factors could represent an effective approach to prevent the mentioned volume loss after adipose tissue transplantation.

At this point it should also be mentioned that in addition to growth factors other substances, in particular derivatives of hyaluronic acid, can have a positive effect on ASCs. It has recently been shown that hyaluronan hybrid cooperative complexes (HCCs)-based formulations could significantly improve the adipogenic differentiation and proliferation of ASCs. Thus, injections of HCCs into the subdermal fat compartment led to an increased recruitment and differentiation of stem cells in adipocytes and thus significantly improved the renewal of the adipose tissue [33].

Previously, we have quantified naturally occurring levels of growth factors in isolated human adipose tissue. We could demonstrate that in cultured adipose tissue growth factor synthesis of bFGF, IGF-1, and VEGF remained nearly stable for several days [34]. Although a positive role of growth factors in regenerative therapy approaches with adipose tissue is undisputed, until up today there is no data on the impact of adipose tissue growth factors concentration on proliferation and migration of its mesenchymal stroma cells (ASC). Therefore, in the present study we have investigated for the first time the relevance of the individual amount of naturally occurring growth factors in adipose tissue on the emigration and proliferation capacity of ASCs. Furthermore, we have evaluated the influence of exogenously applied growth factors on migration and proliferation of ASCs obtained from adipose tissues of donors with different adipose tissue growth factor concentrations.

## Materials and methods

### Material

If not indicated otherwise, all chemicals were obtained from Sigma (Deisenhofen, Germany).

### Samples and preparation

Eight adult white women and seven adult white men, aged 39.9±15.6 years, who had no major systemic metabolic diseases or lipid disorders were enrolled in this study. The ethics committee approved the study plan and all patients gave written informed consent. Lipoaspirates were harvested from the abdomen by a single surgeon (S.R.C.) according to the Coleman protocol [5] and the adipose tissue was transferred directly to the laboratory for further analysis. Briefly, through a small incision at the lower abdominal donor site a mixed solution containing 0.5% lidocaine and 1:200.000 of epinephrine in lactated Ringer's solution was infiltrated by a blunt Lamis infiltrator (Byron Medical. lnc. Tucson. Ariz.). The amount of solution used was identical to the amount of fatty tissue transplant to be harvested. The harvest cannula (blunt tip, 3 mm diameter, 15 or 23 cm in length) was attached to a 10 ml Luer-Lok syringe. By pulling back on the plunger of a 10 ml syringe a slight negative pressure was produced and during fat collection the cannula was advanced and retracted through in the adipose tissue. After filling the syringe with the harvested adipose tissue the cannula was removed and the syringe was sealed with a Luer-Lok stopper. Then the plunger was removed from the syringe and the filled syringe was centrifuged in a centrifuge for 3 minutes at 190 g. After centrifugation the oil layer (top layer) was decanted and the upper aqueous layer was also drained from the syringe. The middle layer which consists predominantly of adipose tissue grafts was studied subsequently [34].

### FACS-analysis of stroma cell phenotype

In order to characterize the stroma cell phenotype of outgrown cells, 8 ml of adipose tissue lipoaspirate were seeded in a 10 cm Petri dish and were maintained for 3 days in 10 ml DMEM supplemented with 10% FBS, 2mM L-Glutamine and 1% Penicillin/Streptomycin at 37°C at 5%CO_2_. Cells emigrated from fat lobules were detached by 0.5% trypsin and 0.02% EDTA, washed (centrifuged at 200 g for 5 min), and stained for 15 min on ice with antibodies against CD14, CD19, CD34, CD45, CD73, CD90, CD105, and HLA-DR for 30 min [35]. Labeled cells were analyzed using a FACSCalibur analyzer (BD biosciences, Heidelberg, Germany).

### Quantification of growth factor content of adipose tissue samples

Growth factor content of 15 individual adipose tissue samples was quantified exactly as described by us previously [34], and protein content-normalized values were depicted in the form of boxplots.

For quantification of growth factor contents of adipose tissues, isolated fat lobules maintained in PBS (containing 10 mM HEPES, 0,5% Triton X-100), protease inhibitor, pH 7.4), were homogenized with an Ultra-Turrax (Polytron PT 1200, Kinematica AG, Luzern, Switzerland) for 30 s, centrifuged at 1,000 g for 10 minutes and at 20,000 g for 10 minutes at 4°C. After discarding the pellet the content of IGF, bFGF, VEGF was quantified in the supernatants using a specific ELISA (R&D Systems, Wiesbaden, Germany) and the Microplate Reader Fluostar OPTIMA (BMG LABTECH GmbH, Ortenberg, Germany) or the MRX II Dynex Technologies Elisa-Reader (Dynex Technologies GmbH, Denkendorf, Germany). Values were normalized to the protein content of the respective sample (DC Protein Assay, Bio-Rad Laboratories GmbH, München, Germany) and were depicted in the form of boxplots, which indicate four quartiles each representing an interval of 25% of the entire data set. As indicated in Fig 4D, the top point of the first quarter of the data points is "Q_1_", and so forth; Q_1_ is also the middle number for the first half of the list, Q_2_ is also the middle number for the whole list, the median Q_3_ is the middle number for the second half of the list, and Q_4_ is the largest value in the list.

### Embedding of fat lobules in collagen gel and visualization of outspreading cells

**Fig 1:**
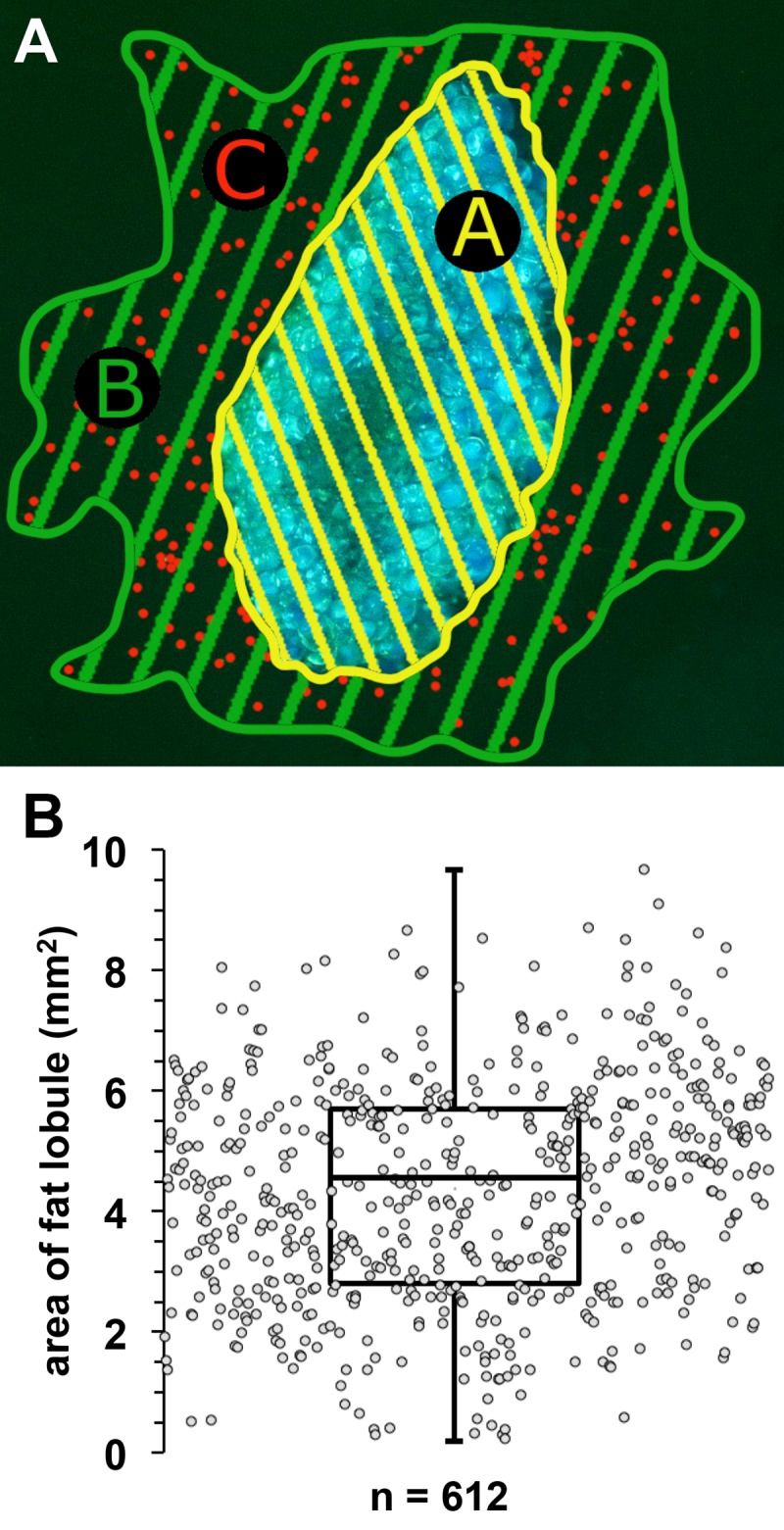
Size distribution of used lobules and index creation. **A,** Schematic outline of collagen-gel embedded fat lobule with outspreading cells. Shown are schematic drawing of the area of cell migration (green B), area of the fat lobule (yellow A) and number of migrated cells (red C). **Index calculation: migration index**: Ratio of area of cell migration (B) and the area of the fat lobule (A); **proliferation-index**: Ratio of number of migrated cells (C) and the area of the fat lobule (A); **Density-index**: Ratio of density of outgrowth (C/B) and the area of the fat lobule. **B**, Size distribution of used lobules.

In order to maintain 3D architecture and an optimally possible physiological environment, washed lobules were embedded in a collagen gel matrix. The size of the used lobules was 4.35±1.82 mm^2^ with a median value of 4.42 mm^2^, which is nearly identical with the lobules mean value of all lobules examined in our experiments (Fig 1B).

Collagen gel was prepared from 0.2% Collagen R Solution (SERVA Electrophoresis, Heidelberg, Germany) mixed with DMEM and NaOH 0.37 M at a ratio of 2:1. To cultivate the lipoaspirate in the collagen gel, 150 μl collagen and 35.5 μl medium/NaOH were injected into the wells of a 24-well plate. In each case 6–10 lobules of each patient were inserted. In order to ensured that the lobes were securely immobilized and adhesion was ensured, a new layer of collagen gel was layered over the lobules [36, 37]. The upper layer of gel was then coated with 1 ml preadipocyte medium composed of 20 ml FCS, 2 ml Penstrep and 180 ml DMEM/HAMS.

Vital cells were visualized by using fluorescein diacetate (FDA, final concentration 50 μg/ml). Cell nuclei were visualized by using the Hoechst dye H33342 (final concentration 25 μg/ml). Staining was evaluated using fluorescence microscopy (extinction at 480 nm, emission at 520 nm) and H33342-staining was evaluated using a 350 nm extinction filter and 461 nm emission filter.

The amount of emigrated cells and the area they covered after three days was documented by photography (Fig 1A).

### Evaluation of cell migration, proliferation, and density

In each case of the 15 individual adipose tissue samples 6–10 individual fat lobules were embedded in the collagen gel as described above. With these fat lobules we have evaluated the outgrowth, proliferation, and migration potency of multi-potent stromal cells (MSCs) and have correlated the obtained values with the growth factor concentration of the adipose tissue samples the lobules were isolated from. A graphical outline of the experimental setup is shown in S1 Fig.

For a better understanding of the regenerative potential of isolated fat lobules we calculated three indices based on number and/or the migration distance (settled area) of outgrown cells in dependence to the size of the particular fat lobules. The size of the fat lobules, number of outgrown cells and the emigration area was evaluated from single photographs of stained samples using the ‘Discus’ software (Diskus-Mikroskopische Diskussion, Carl H. Hilgers-Technisches Büro, Königswinter, Germany) as exemplarily documented in Fig 1A.

#### Migration index

Ratio of area of cell migration (area B) and the area of the fat lobule (area A) (B/A in Fig 1A).

#### Proliferation index

Ratio of number of migrated cells (indicated as C) and the area of the fat lobule (area A) (C/A in Fig 1A).

#### Density index

Ratio of density of outgrowth (C/B) and the area of the fat lobule ((C/B)/A in Fig 1A).

### Impact of exogenously added growth factors on proliferation, migration and density of cells outgrown from fat lobules

In order to evaluate the impact of exogenously added growth factors on proliferation and migration of cells from fat lobules, collagen-embedded fat lobules of each donor sample, as described above, were cultured in the absence or additionally in the presence of recombinant human growth factors: IGF (50 ng/ml), bFGF (3.45 ng/ml), or VEGF (100 ng/ml). Samples with the collagen embedded fat lobules were maintained for three days in 1 ml growth medium (DMEM/HAMS, 10% FCS, 0.5% Penstrep). After three days migration and proliferation indices were calculated as mentioned above and again were correlated to values of growth factor contents of the original adipose tissue the lobules were isolated from.

### Impact of supraphysiological growth factor concentration on MSC outgrowth from fat lobules

To further characterize the impact of adipose tissue growth factor content on the outgrowth potential of its MSCs we have cultivated freshly isolated adipose tissue samples (approx. 5 x 5 x 5 mm cubes) for seven days in DMEM plus 2.5% FCS in the absence or presence of a growth factor mix at supraphysiological concentrations consisting of 200 ng/ml IGF plus 50 ng/ml bFGF plus 300 ng/ml VEGF. Alternatively, adipose tissue samples were maintained for seven days in strongly growth factor reduced cell culture medium (DMEM plus 2.5% FCS). In both cases we used adipose tissue samples with low growth factor contents belonging to the fist quartile (Q1) or high growth factor concentration belonging to the fourth quartile (Q4). After the respective treatment procedure adipose tissue samples were digested as described above and 50 lobules per 10 cm cell culture Petri dish were seeded and maintained in 10 ml DMEM supplemented with 10% FBS, 2 mM L-Glutamine and 1% Penicillin/Streptomycin at 37°C at 5%CO_2_. Alternatively, the medium was supplemented with a growth factor mix consisting of IGF (50 ng/ml) plus bFGF (3.45 ng/ml) plus VEGF (100 ng/ml). After 3 days of cultivation the outgrown cells were detached by 0.5% trypsin and 0.02% EDTA, washed, centrifuged at 200 g for 5 min, and quantified using a Neubauer chamber.

### Statistical analysis

Significant differences were evaluated using either Wilcoxon test, paired two-tailed Student's t-test or ANOVA followed by an appropriate post-hoc multiple comparison test (Tukey method). A *p*<0.05 was considered statistically significant.

### Ethics approval and consent to participate

The experimental protocol and the use of human material have been approved by the local ethics committee of the Medical Faculty of the RWTH University Aachen (votum number: EK163/07) as well as the Faculty of Medicine of the Heinrich-Heine-University Düsseldorf (study number: 3634). All experiments were conducted in compliance with the Declaration of Helsinki Principles.

## Results

### Evaluation of the stroma cell character of cells grown out from isolated fat lobules

The FACS-based phenotypic analysis of the outgrown cells showed that the cells were positive for the expression of CD73, CD90, and CD105 but negative for the expression of CD14, CD19, CD34, CD45, and HLA-DR (S2 Fig). Thus the cells revealed a significant mesenchymal stroma cell phenotype which comply with the criteria of the International Society of Cellular Therapy for mesenchymal stem cells [35].

### Isolated fat lobules

As indicated in the *Material and Method* section, the size of lobules strongly influences the value of the calculated migration, proliferation and density indices. Therefore, for a better comparison of the results we have sought for all of our experiments to use lobules of comparable size (S3 Fig). In the present study we have calculated the proliferation, migration, and density indices on the basis of in total 612 fat lobules with an average lobule-size of 4.35±1.82 μm^2^ (Fig 1B).

### Growth factor content of isolated adipose tissues samples and impact of autologous growth factors on outgrowth, migration and proliferation of fat lobules-derived mesenchymal stroma cells

**Fig 2:**
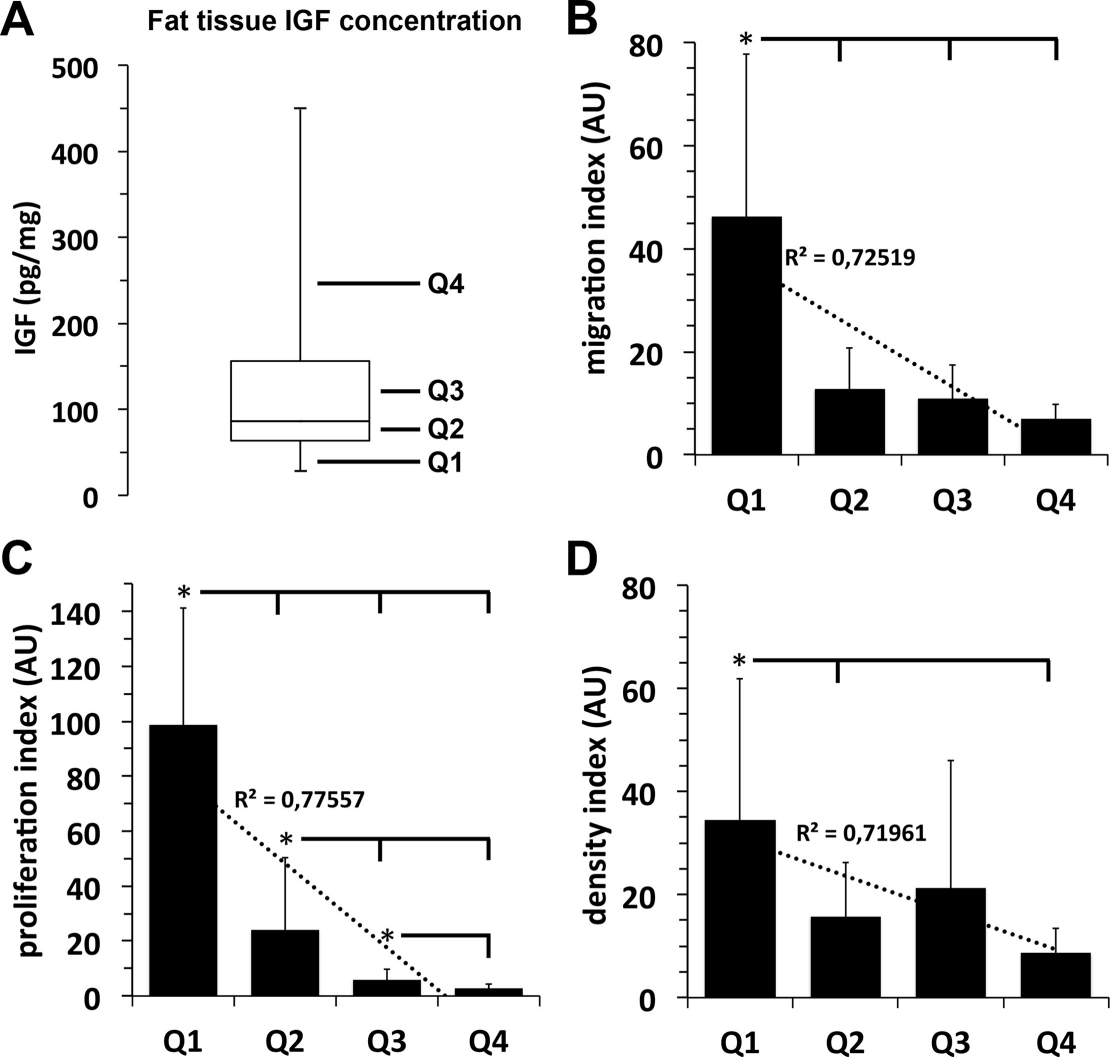
Correlation between adipose tissue sample IGF concentration and the migration and proliferation capacity of its mesenchymal stroma cells. **A**, For quantification of the individual physiological adipose tissue growth factor content, freshly isolated adipose tissue samples were homogenized and the amount of IGF was quantified by specific ELISAs. IGF concentration values of 15 individual adipose tissue samples are shown as boxplot with whiskers with minimum and maximum. Quartiles (25% of values) are indicated as Q1, Q2, Q3, and Q4. **B–D:** Migration capacity (**B**), proliferation capacity (**C**), and density index (**D**) of MSCs that grew out of fat lobules which were isolated from adipose tissue containing IGF values of the respective concentration quartiles Q1, Q2, Q3 or Q4. *, *p*<0.05 as compared to values indicated.

**Fig 3:**
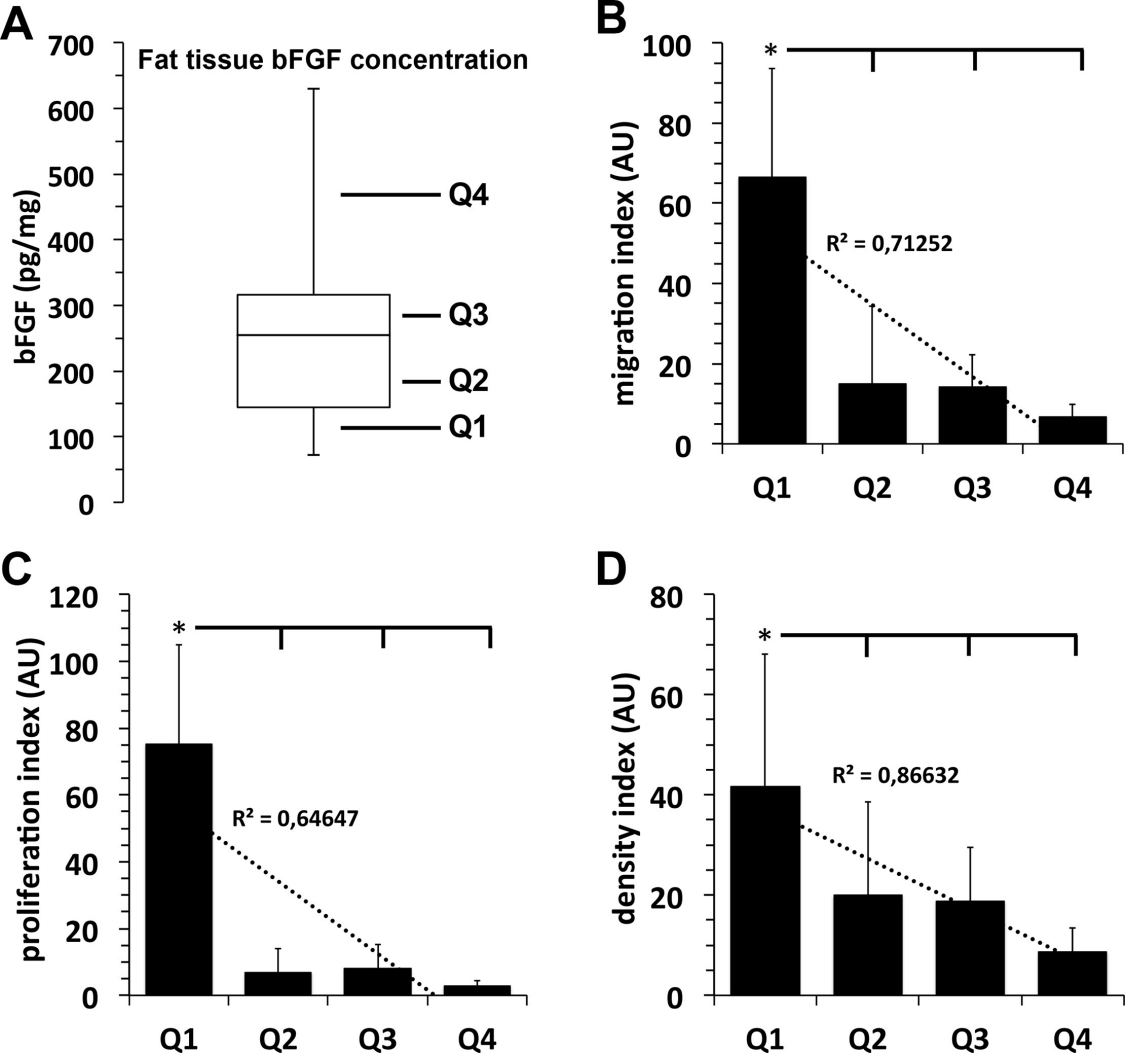
Correlation between adipose tissue sample bFGF concentration and the migration and proliferation capacity of its mesenchymal stroma cells. **A**, For quantification of the individual physiological adipose tissue growth factor content, freshly isolated adipose tissue samples were homogenized and the amount of bFGF was quantified by specific ELISAs. bFGF concentration values of 15 individual adipose tissue samples are shown as boxplot with whiskers with minimum and maximum. Quartiles (25% of values) are indicated as Q1, Q2, Q3, and Q4. **B–D:** Migration capacity (**B**), proliferation capacity (**C**), and density index (**D**) of MSCs that grew out of fat lobules which were isolated from adipose tissue containing bFGF values of the respective concentration quartiles Q1, Q2, Q3 or Q4. *, *p*<0.05 as compared to values indicated.

**Fig 4:**
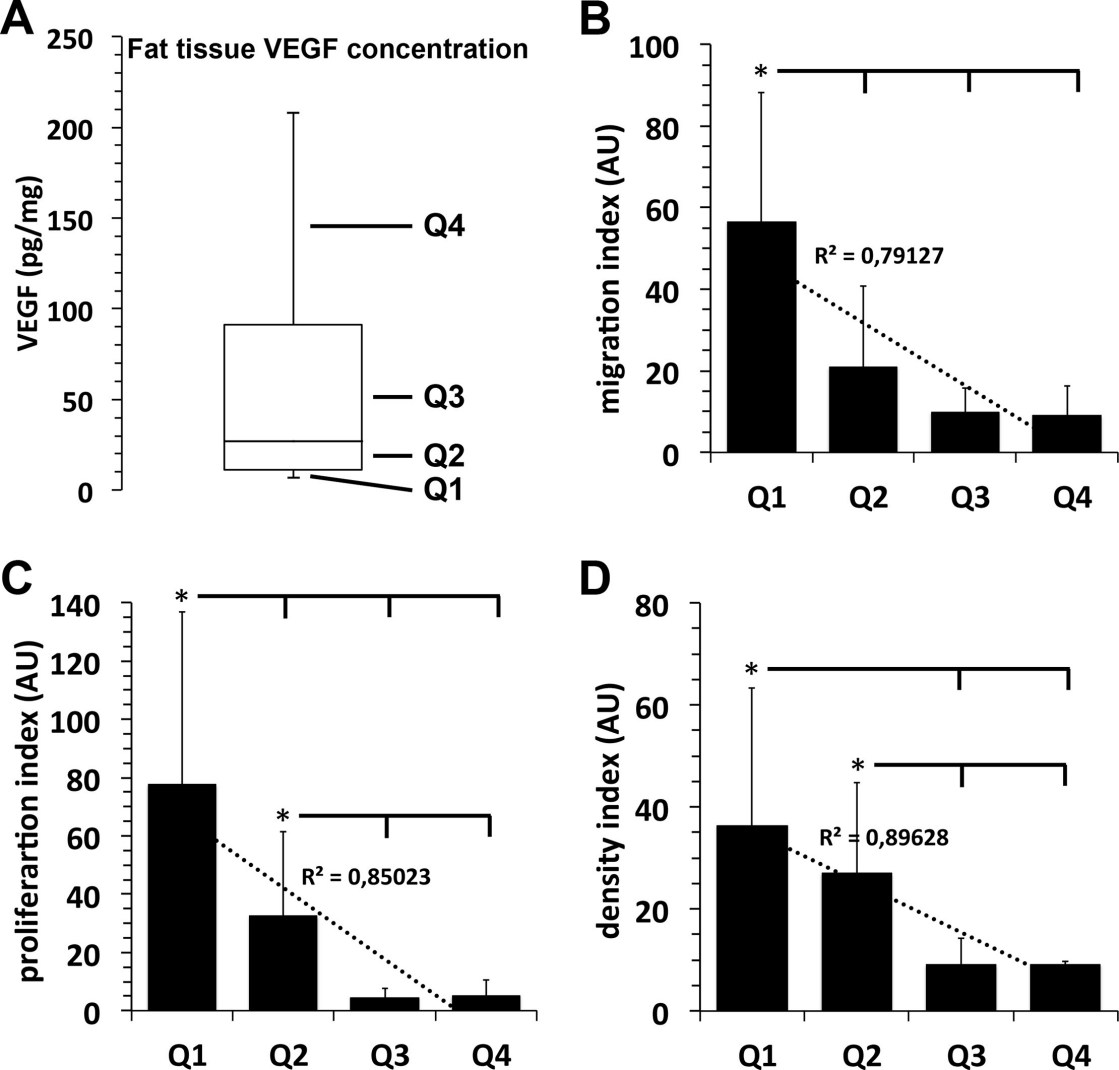
Correlation between adipose tissue sample VEGF concentration and the migration and proliferation capacity of its mesenchymal stroma cells. **A**, For quantification of the individual physiological adipose tissue growth factor content, freshly isolated adipose tissue samples of 15 individuals were homogenized and the amount of VEGF was quantified by specific ELISAs. VEGF concentration values of the 15 adipose tissue samples are shown as boxplot with whiskers with minimum and maximum. Quartiles (25% of values) are indicated as Q1, Q2, Q3, and Q4. **B–D:** Migration capacity (**B**), proliferation capacity (**C**), and density index (**D**) of MSCs that grew out of fat lobules which were isolated from adipose tissue containing VEGF values of the respective concentration quartiles Q1, Q2, Q3 or Q4. *, *p*<0.05 as compared to values indicated.

In the examined adipose tissue samples we observed strong inter-individual differences in growth factor content upon the examined donor population. IGF was found in the concentration range of 25–450 pg/mg protein with a median at 80 pg/mg protein (Fig 2A), bFGF at concentrations of 60–630 pg/mg protein with a median at 250 pg/mg protein, (Fig 3A), and VEGF was detected at concentrations of 7–215 pg/mg protein with a median at 27 pg/mg protein (Fig 4A).

Furthermore, results shown in Fig 2B–2D reveal an apparent and significant negative correlation between IGF concentrations of adipose tissue and the migration (Fig 2B) and proliferation capacity (Fig 2C) of mesenchymal stroma cells that have grown out of the respective adipose tissue samples. Highest migration and proliferation rates of fat lobule-derived mesenchymal stroma cells were observed with fat lobules obtained from adipose tissue with the 25% lowest values of IGF concentration, values of the first quartile (Q1) in Fig 2A.

In contrast, lowest migration and proliferation rates of fat lobule-derived mesenchymal stroma cells were observed with fat lobules obtained from adipose tissue with the 25% highest values of IGF concentration, values of the fourth quartile (Q4) in Fig 2A.

Analogous to these results, we observed an identical inverse correlation between the adipose tissue concentration of bFGF (Fig 3) or VEGF (Fig 4) and the proliferation and migration rate of ASCs that have grown out of these adipose tissue samples.

**Fig 5:**
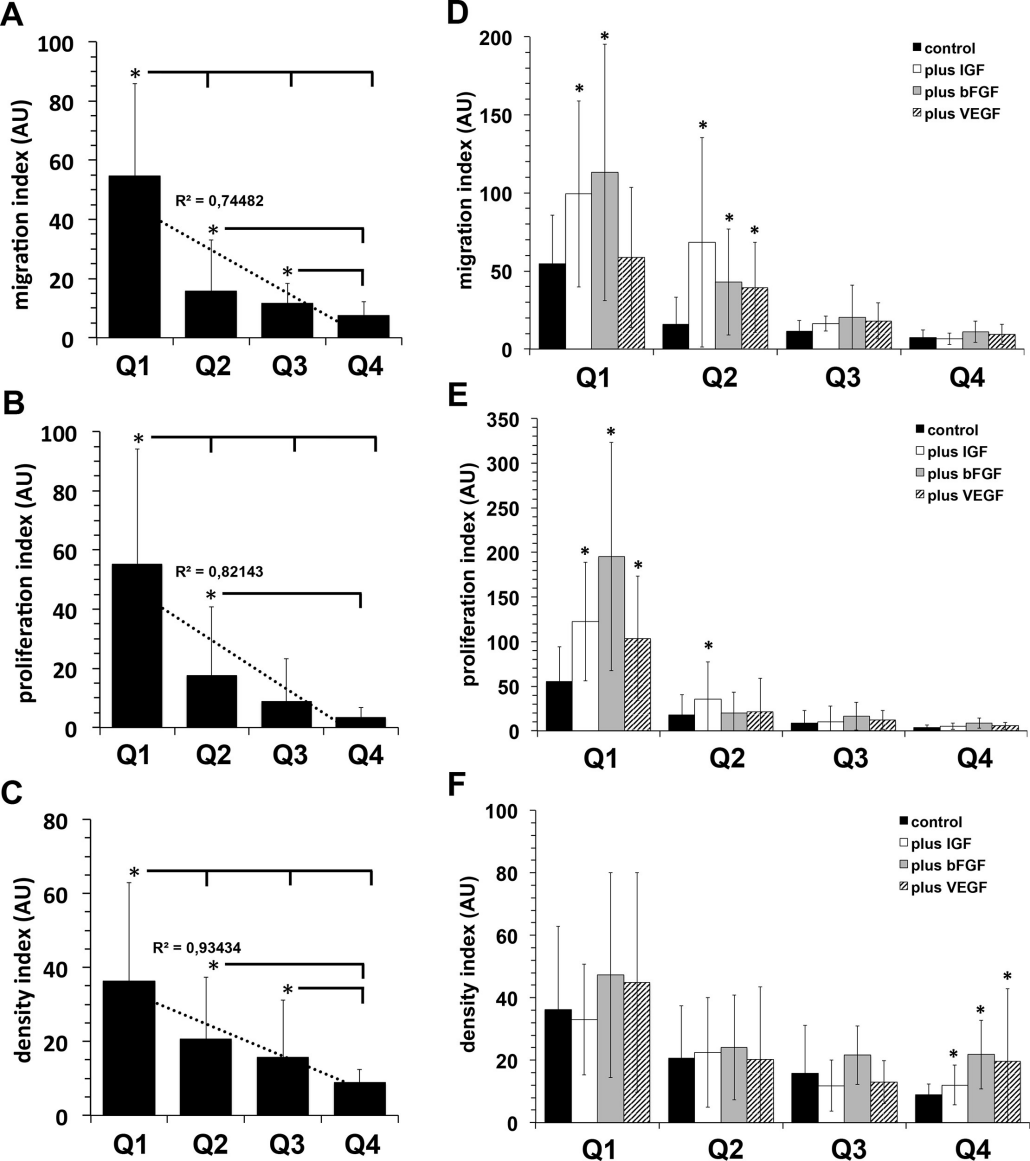
Impact of adipose tissue growth factors content or exogenously added growth factors on outgrowth, migration and proliferation capacity of adipose tissue-located mesenchymal stroma cells. In order to further estimate the impact of growth factors on migration and proliferation capacity of adipose tissue-resident mesenchymal stroma cell, we have integrated all related raw data of Q1—Q4 shown in Figs 2–4. **A**, Migration capacity. **B**, Proliferation capacity. **C**, Density index. Values represent mean ± SD. *, *p*<0.05 as compared to values indicated. **D–E,** In order to evaluate the impact of growth factors supplementation on proliferation and migration of mesenchymal stroma cells from fat lobules used in Figs 2–4, gelatin embedded fat lobules were cultivated for three days with growth medium additionally containing recombinant human IGF (50 ng/ml), bFGF (3.45 ng/ml), VEGF (100 ng/ml) or a mix of all three growth factors. After three days the indices reflecting migration and proliferation capacity were calculated and correlated to growth factor contents of the particular used adipose tissue. Finally, all related raw data were pooled referring the procedure mentioned above for **A–C**. **D**, Migration capacity. **E**, Proliferation capacity. **F**, Density index. Black bars, control values (identical to the respective values shown in **A–C**); white bars, incubation with recombinant human IGF; gray bars, incubation with recombinant human bFGF; striped bars, incubation with recombinant human VEGF; vertical striped bars, incubation with a mix of the three growth factors. Values represent mean ± SD. *, *p*<0.05 as compared to control values of the respective quartile.

In order to further support the significance of results described above we pooled all growth factor-relating raw data of Figs 2–4. Thus, Fig 5A contains the summarized growth factor dependent proliferation data of Figs 2B, 3B and 4B, Fig 5B contains the summarized growth factor dependent migration data of Figs 2C, 3C and 4C, and Fig 5C contains the summarized growth factor dependent density index data of Figs 2D, 3D and 4D. This form of presentation clearly underline our observation of a reciprocal correlation between proliferation and migration capacity of adipose tissue derived mesenchymal stroma cells and the growth factor content of the origin adipose tissue sample.

### Impact of exogenously added growth factors on proliferation, migration and density of cells outgrown from fat lobules

In order to mimic the possible influence of an additional therapeutic administration of exogenous growth factors and to characterize their influence on the growth and migration of adipose tissue-derived ASCs, we cultivated gelatin embedded fat lobules, that otherwise were identically treated as shown in Figs 2–4, for three days in the presence of recombinant human IGF (50 ng/ml), bFGF (3.45 ng/ml), or VEGF (100 ng/ml) or a mixof these thre growth factors. After three days the indices reflecting migration and proliferation capacity were calculated and correlated to growth factor contents (IGF, bFGF, VEGF in accordance to Figs 2A, 3A and 4A) of the particular adipose tissue. Then, all growth factor-related raw data were pooled referring to the procedure described for Fig 5A–5C.

Results summarized in Fig 5D–5F show that exogenously applied growth factors, regardless of whether administered alone or as a mix of the three growth factors, significantly enhanced migration of mesenchymal stroma cells of fat lobules that were isolated from adipose tissue with the lowest growth factor concentrations (values of the first quartile Q1 in Figs 2A, 3B and 4C). In contrast, exogenously applied growth factors did not have any significant effects on migration and proliferation capacity of mesenchymal stroma cells of fat lobules obtained from adipose tissue with growth factor production values of the 50% highest rankings of Q3 and Q4 shown in Figs 2A, 3A and 4A.

### High growth factor concentration significantly affects MSCs outgrowth from fat lobules

**Fig 6:**
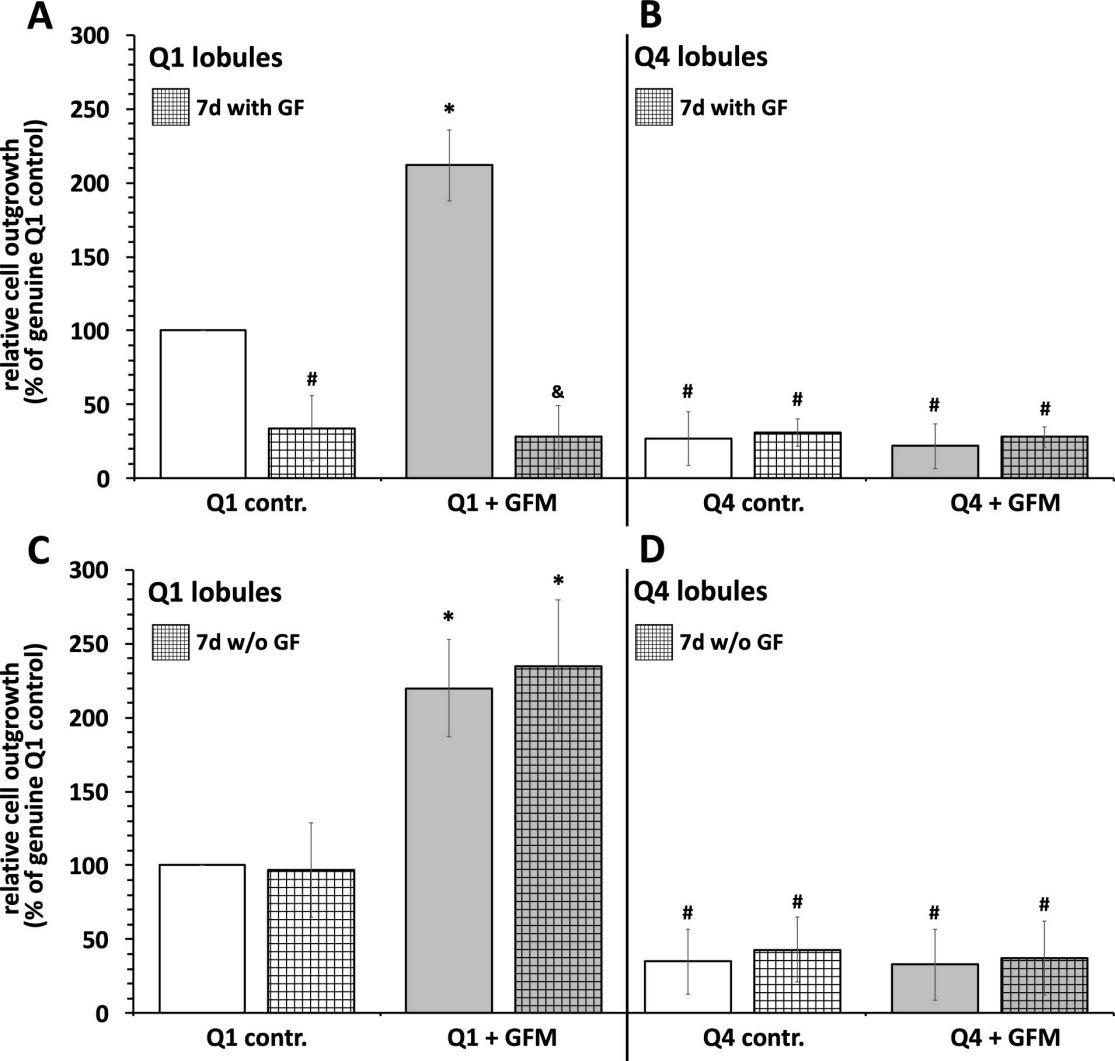
High environmental growth factor concentration significantly reduced MSCs outgrowth from fat lobules. Freshly isolated adipose tissue samples (approx. 5 x 5 x 5 mm cubes) obtained from adipose tissue samples with low growth factor concentrations (**Q1 lobules**) or high growth factor concentration (**Q4 lobules**) were maintained for seven days in cell culture medium containing supraphysiological concentrations of growth factors (**A and B**: **GF**; 200 ng/ml IGF plus 50 ng/ml bFGF plus 300 ng/ml VEGF; squared bars). Alternatively, adipose tissue samples were maintained for seven days in growth factor-reduced cell culture medium, squared bars in **C and D**. After digestion of the adipose tissue 50 lobules were seeded on each of 10 cm cell culture Petri dishes and were maintained for three days in the absence (white bars) or presence (gray bars) of a growth factor mix (**GFM;** 50 ng/ml IGF plus 3.45 ng/ml bFGF plus 100 ng/ml VEGF) identical as described in Fig 5. After 3 days of cultivation cells that grew out of the lobules were quantified. **A and B**, MSC outgrowth from lobules preincubated for seven days with supraphysiological concentrations of growth factors (squared bars, **7d with GF). A**, relative number of MSCs that grew out from adipose tissue samples with low growth factor content (**Q1 lobules**). **B**, relative number of MSCs that grew out from adipose tissue samples with high growth factor content (**Q4 lobules**). **C and D**, MSC outgrowth from lobules maintained for seven days under growth factor depleted conditions (squared bars, **7d w/o GF). C**, relative number of MSCs that grew out from adipose tissue samples with low growth factor content (**Q1 lobules**). **D**, relative number of MSCs that grew out from adipose tissue samples with high growth factor content (**Q4 lobules**).

As shown in Fig 6A and 6B, MSCs from adipose tissue with low growth factor contents (Q1 lobules) exhibited a significantly higher outgrowth potential than MSCs from adipose tissue with high growth factor contents (Q4 lobules). Exactly as already shown in Fig 5, MSC outgrowth from Q1 lobules but not of Q4 lobules could be significantly enhanced by an exogenously applied growth factor mix. In contrast, with Q1 lobules that were preincubated for seven days with supraphysiological high growth factor concentration the mentioned pronounced basal as well as growth factor-induced enhancement of MSC outgrowth was significantly reduced. The growth behavior of MSCs from the Q4 lobules was in no way influenced by the aforementioned seven days lasting pre-incubation with growth factors. Furthermore, prolonged incubation of the Q4 lobules in a nearly growth factor free environment also had no effect on the growth behavior of the Q4 MSCs (Fig 6D).

## Discussion

The use of autologous adipose tissue grafts has many advantages in the reconstruction of soft tissue volume as well as contour defects [38]. However, the successful use of fat grafting is often limited by the sometimes low and often unpredictable survival rates [39, 40]. As a result of the often-unsatisfactory results of adipose tissue grafts various experimental and clinical attempts have been made to increase survival rates of the implanted adipose tissue. For this purpose the transferred adipocytes were exposed to a more supportive biochemical environment, e. g. by adding growth factors [11]. Some reports are indicative of the pivotal role of the naturally occurring levels of growth hormones in transplanted adipose tissue [11, 41] as well as growth factor production of preadipocytes and its contribution to a successful outcome of adipose tissue transplantation [31, 42]. Indeed, as shown by us previously, freshly isolated lipoaspirates show a considerable amount of IGF-1 and bFGF, and smaller quantities of VEGF [34].

It is well known that Insulin and IGF-1 stimulate adipocyte proliferation, differentiation, and trophic activities [43–46]. In addition, bFGF can stimulate preadipocytes and fibroblasts on two levels, directly as mitogens for mesenchymal cells and indirectly as angiogenic factor [47–51]. bFGF and VEGF can significantly accelerate the graft revascularization process and increase the vascular architecture of the grafted tissue. As a result, both growth factors can create an environment in which existing tissue is better supported and preadipocyte proliferation and differentiation is specifically promoted. Therefore, long term delivery of insulin, IGF-1, and bFGF is expected to successfully increase the weight maintenance of subdermally implanted autologous adipose tissue grafts [52]. All these findings imply a positive correlation between the growth factor content of the adipose tissue sample and structural adipose tissue acceptance at the donor site. Thus, significant inter-individual differences in growth factor levels as measured by us previously [34] might explain reduced volume retention in some patients.

In the present study we affirm our previous observation of strong inter-individual differences in adipose tissue growth factor concentration upon the examined donor population. Thus we found an adipose tissue IGF concentration of 25–450 pg/mg protein, bFGF of 60–630 pg/mg protein, and VEGF of 7–215 pg/mg protein. Interestingly and in contrast to our expectations, we found an inverse reciprocal correlation between the detected values of growth factor concentration of the characterized human adipose tissue samples and the proliferation and migration capacity of mesenchymal stroma cells (MSCs) obtained from the examined adipose tissue samples. Apparently paradoxical, we observed highest migration and proliferation values with mesenchymal stroma cells from adipose tissue containing the lowest values of growth factor content, whereas lowest migration and proliferation capacity of resident mesenchymal stroma cells could be found with fat lobules obtained from adipose tissue with the highest growth factor content.

In the last 10 years, the proliferation-increasing role of growth factors as well as their role in increasing the survival rate of MSCs has been extensively studied. The effect of most growth factors is pleiotrophic so that they can affect several biological factors simultaneously, e.g. motility, proliferation, morphogenesis and survival [53]. This is the reason why growth factors in regenerative medicine are frequently used to circumvent problems of MSC proliferation and expansion as well as survival *in vitro* and *in vivo*. Due to their synergistic effect, treatments with combinations of different growth factors are currently receiving particular attention.

Nevertheless, the wide-ranging biological roles of growth factors and the multitude of signaling pathways activated by them suggest that in the cell growth factor signaling must be tightly regulated, and positive but also negative feedback loops have been discovered in these pathways. Exemplarily, circulating IGF binds to its receptor (IGF-1R), which leads to the activation of Ras and AKT, two factors that directly lead to the up-regulation of genes involved in cell proliferation, survival, invasion, and angiogenesis [54]. However, AKT is also an upstream regulator of the mammalian target of rapamycin complex 1 (mTORC1) and a downstream effector of mTORC2, whereby both mTOR variants playing an important role in positive and negative feedback on the IGF/AKT signaling pathway. While mTORC1 represents an indirect inhibitor of AKT activity [55], mTORC2 acts as an effective AKT activator [56].

The FGF signaling cascade can be regulated at multiple levels by negative feedback mechanisms through the proteins Dual Specificity Phosphatases (DUSPs) and Spred and Sprouty [57–59]. In this case, the negative feedback mechanism mediated by DUSPs, Spred and Sprouty leads to the reduction or termination of the FGF signaling pathway [60]. Active FGF signaling thus promotes both a primed state and the expression of inhibitors of FGF signaling to create a negative feedback loop that would facilitate the return to the original unprimed state [60].

VEGF is one of the most important angiogenic factors. The biological effects of VEGF are mediated at the cellular level by its two membrane receptors VEGFR-1 and VEGFR-2 [61]. However, there is also a soluble form of the receptor, sVEGFR-1, which can bind the free form of VEGF-A and thus acts as a potent anti-angiogenic factor [62]. In addition, because VEGF-A has been shown to upregulate sVEGF-R1 expression but does not affect the expression of VEGF-R1, such a VEGF-A mediated sVEGFR-1 upregulation can be considered as a negative feedback system of VEGF action [63].

Concerning MSC proliferation and migration our results indicate a negative conditioning by higher growth factor concentrations in the MSC environing tissue. The assumption of a growth factor-dependent negative feedback mechanism on MSC obtained from adipose tissue containing elevated growth factor concentrations is further supported by our finding that exogenously applied recombinant growth factors did not have any significant effects on migration and proliferation capacity of those cells. In contrast, with fat lobules obtained from adipose tissue containing the lowest values of growth factor additional supplementation with recombinant growth factors led to significantly enhanced proliferation and migration of adipose tissue-derived mesenchymal stroma cells. Another convincing argument for our assumption described above is our observation that the high proliferation potential of cells from adipose tissue with low GF content (Q1) could be significantly reduced by a prolonged preincubation of the adipose tissue with high GF concentrations. The growth behavior of the Q1 MSCs was almost identical to the proliferation- impaired MSCs from adipose tissue with a very high GF content (Q4 lobules). So here we can give further indications that an exogenous GF environment can significantly prime the proliferation behavior of adipose tissue MSCs.

Our study shows for the first time that the capacity of mesenchymal stroma cells to spread out of isolated fat lobules as well as their proliferative potency depends inversely proportional on the growth factor content of the considered adipose tissue. Furthermore, our findings demonstrate that recombinant growth factors were inefficient to enhance MSC proliferation and migration in adipose tissue samples exhibiting high growth factor contents. This indicates a growth factors-mediated negative feedback mechanism leading to an impaired growth factor answer of MSC obtained from adipose samples containing high growth factor concentration. Therefore, growth factor content of adipose tissue could serve as a predictive clinical parameter for the therapeutic outcome of autologous adipose tissue transfer. Our results implicate that in the case of low growth factor concentrations of the isolated adipose tissue a better therapeutic outcome of the adipose tissue graft could be expected. In this case, the attending physician would need to transplant less fatty tissue to get a good result. Additionally, in this case an additional supplementation with exogenously applied growth factors would make sense and even would improve the therapeutic outcome. In contrast, in the case of high growth factor concentration in the isolated adipose tissue sample, due to the lower proliferation and emigration capacity of the MSCs, it would be necessary to transplant a higher amount of adipose tissue material to achieve a comparable good result as mentioned above in the case of the first scenario. Concerning the clinical outcome however, supplementation with exogenous growth factors would make no sense and one would avoid additional burdening the patient with growth factors.

## Supporting information

S1 Fig Experimental setup. In 15 freshly isolated individual adipose tissue samples we have quantified the amount of the three relevant growth factors IGF, bFGF, and VEGF. Additionally, in 4–10 single lobules, obtained from each of the fat samples, we have evaluated the outgrowth, proliferation, and migration potency of adipose-derived stem cells (ADSCs) and have correlated the obtained values with the growth factor concentrations of the corresponding fat tissue samples. Additionally, in an identical parallel experiment approach we have examined the impact of exogenously applied recombinant human growth factors (rh-IGF, rh-bFGF, rh-VEGF) on outgrowth, proliferation, and migration potency of ADSCs from the collagen-embedded lobules. Again, the obtained results were correlated with the growth factor concentrations of the corresponding fat tissue samples.
(TIF)

S2 Fig Evaluation of the stem cell character of cells grown out from isolated fat lobules. In order to characterize the stem cell phenotype of outgrown cells, adipose tissue lipoaspirates were maintained for 5 days in culture. ADSCs that were grown out from fat lobules were detached by trypsin, stained with antibodies against CD14, CD19, CD34, CD45, CD73, CD90, CD105, and HLA-DR, and the cell surface antigenic phenotype was analyzed using the FACSCalibur analyzer. Values represent mean ± SD of 4–6 individual experiments.
(TIF)

S3 Fig Size control of included fat lobules. Size (projection surface in mm^2^) of lobules obtained from human fat tissue with growth factor concentrations (IGF, FGF, VEGF) corresponding to Q1 –Q4 as indicated in **A** of Figs 2, 3 or 4, respectively. Values represent mean ± SD of 40 lobules.
(TIF)

S1 Data (XLSX)

S2 Data (XLSX)

S3 Data (XLSX)

S4 Data (XLSX)

S5 Data (XLSX)

S6 Data (XLSX)

S7 Data (XLSX)

S8 Data (XLSX)
