# Supplementary figures and images for: Impact of growth factor content on proliferation of mesenchymal stromal cells derived from adipose tissue
Source: PLoS One. 2020 Apr 16;15(4):e0230265. doi: 10.1371/journal.pone.0230265 (PMC7162516; doi:10.1371/journal.pone.0230265)

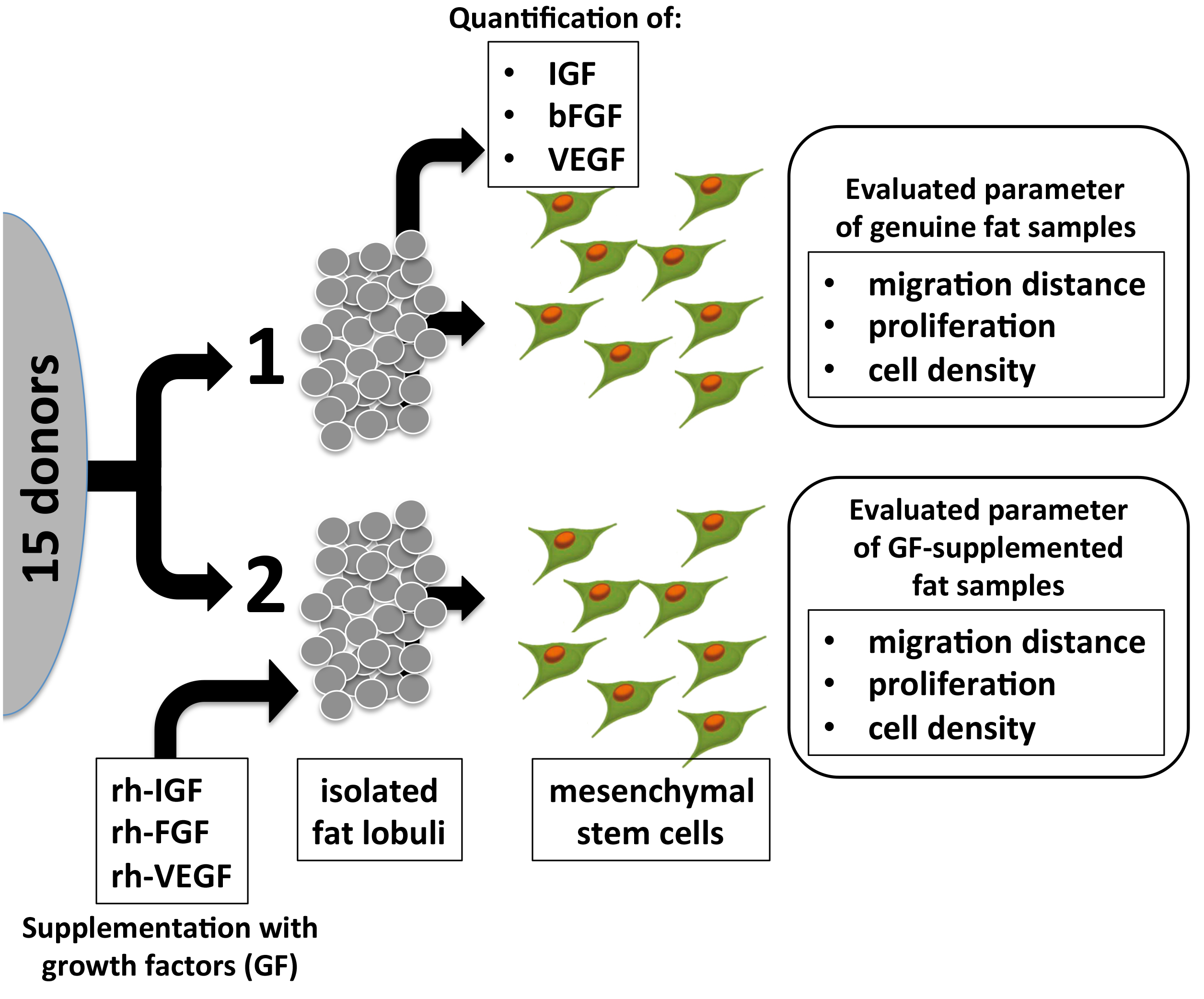

Supplement: S1 Fig — In 15 freshly isolated individual adipose tissue samples we have quantified the amount of the three relevant growth factors IGF, bFGF, and VEGF. Additionally, in 4–10 single lobules, obtained from each of the fat samples, we have evaluated the outgrowth, proliferation, and migration potency of adipose-derived stem cells (ADSCs) and have correlated the obtained values with the growth factor concentrations of the corresponding fat tissue samples. Additionally, in an identical parallel experiment approach we have examined the impact of exogenously applied recombinant human growth factors (rh-IGF, rh-bFGF, rh-VEGF) on outgrowth, proliferation, and migration potency of ADSCs from the collagen-embedded lobules. Again, the obtained results were correlated with the growth factor concentrations of the corresponding fat tissue samples. (TIF) [file pone.0230265.s001.tif]

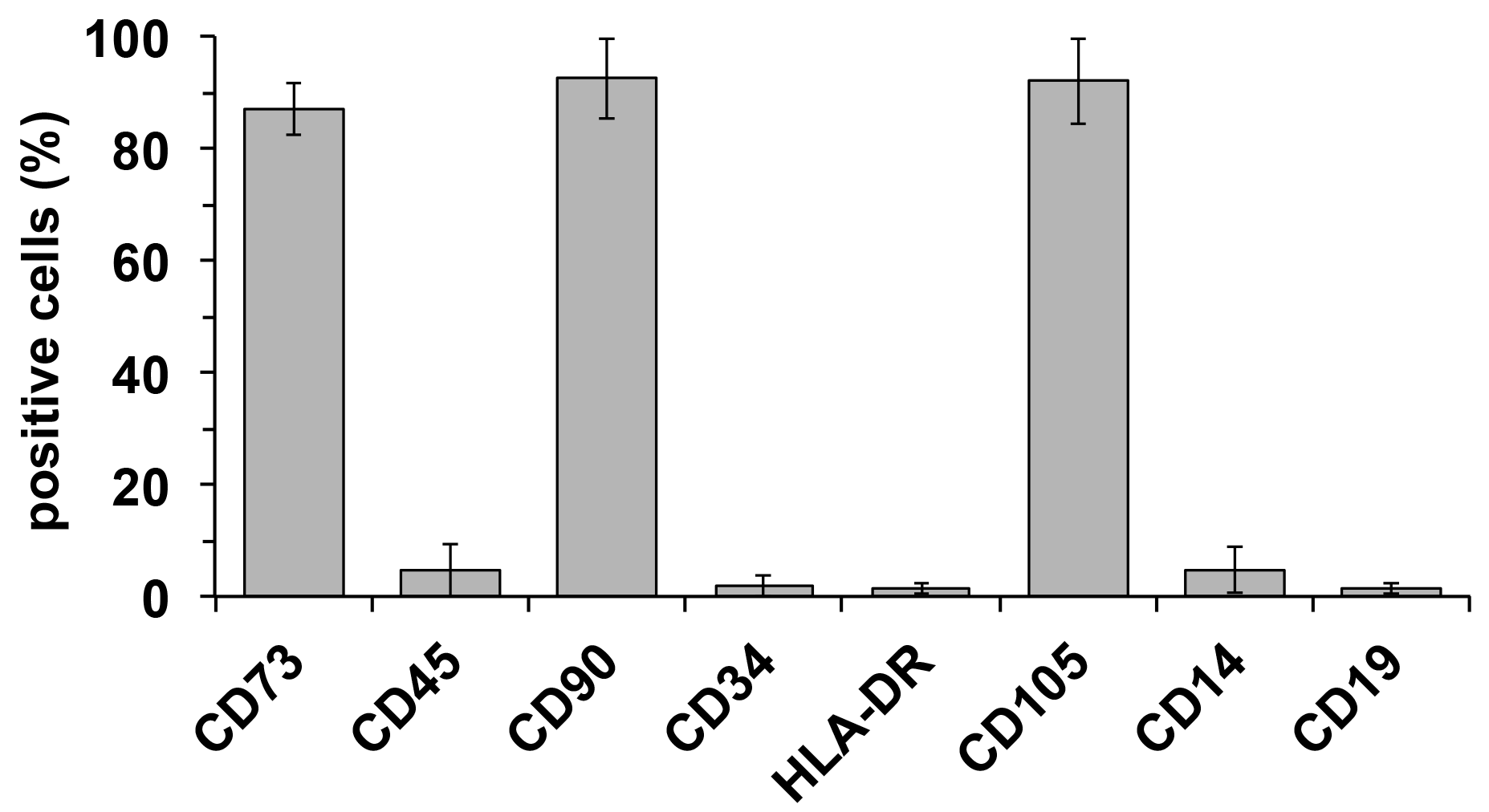

Supplement: S2 Fig — In order to characterize the stem cell phenotype of outgrown cells, adipose tissue lipoaspirates were maintained for 5 days in culture. ADSCs that were grown out from fat lobules were detached by trypsin, stained with antibodies against CD14, CD19, CD34, CD45, CD73, CD90, CD105, and HLA-DR, and the cell surface antigenic phenotype was analyzed using the FACSCalibur analyzer. Values represent mean ± SD of 4–6 individual experiments. (TIF) [file pone.0230265.s002.tif]

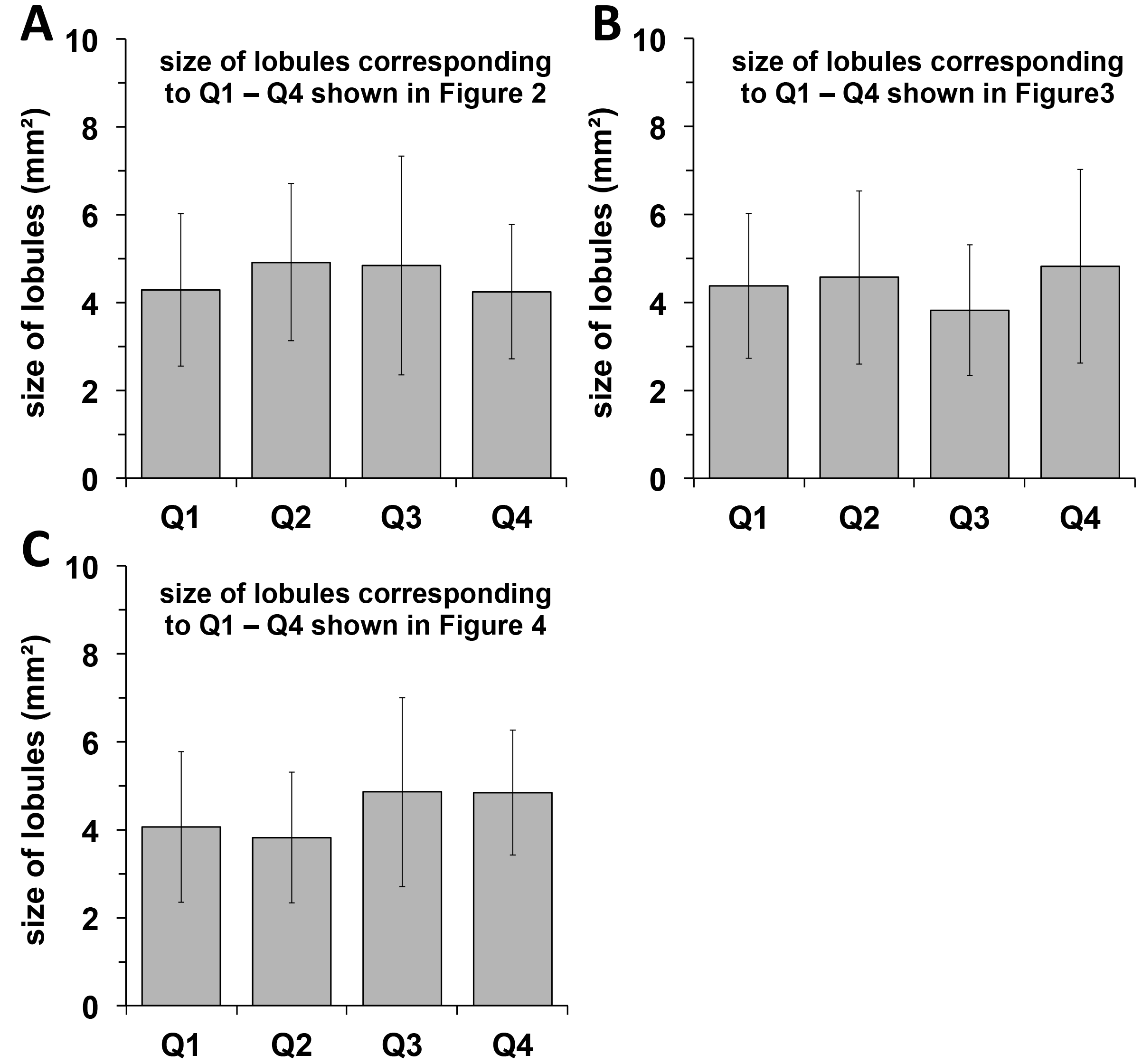

Supplement: S3 Fig — Size (projection surface in mm2) of lobules obtained from human fat tissue with growth factor concentrations (IGF, FGF, VEGF) corresponding to Q1 –Q4 as indicated in A of Figs 2, 3 or 4, respectively. Values represent mean ± SD of 40 lobules. (TIF) [file pone.0230265.s003.tif]
